# Expression, purification of metallothionein genes from freshwater crab (*Sinopotamon yangtsekiense*) and development of an anti-metallothionein ELISA

**DOI:** 10.1371/journal.pone.0174482

**Published:** 2017-03-28

**Authors:** Jian Yang, Hui Sun, Hao Zhang, Hui Zhou

**Affiliations:** 1 Translational Medicine Research Center, Shanxi Medical University, Taiyuan, Shanxi Province, People’s Republic of China; 2 Institute of Science & Technology of Shanxi, Taiyuan, Shanxi Province, People’s Republic of China; Duke University School of Medicine, UNITED STATES

## Abstract

Using the phoA-fusion technology, the recombinant metallothionein (MT) from freshwater crab (*Sinopotamon yangtsekiense*) has been successfully produced in *Escherichia coli*. MT purified from the bacterial suspension showed one polypeptide with a molecular weight of 7 kDa by tricine-sodium dodecyl sulfate-polyacrylamide gel electrophoresis (Tricine-SDS-PAGE). Western-blotting confirmed the polypeptides had a specific reactivity with mouse polyclonal MT anti-serum. Based on the purified MT and MT anti-serum, the reaction parameters for an enzyme-linked immunosorbent assay (ELISA) were developed. The direct coating ELISA showed a higher linear relationship compared to antibody sandwich coating ELISA. The optimal dilution rates of purified MT anti-serum and coating period were shown to be 1:160,000 and 12 hours at 4°C. At 37°C, the appropriate reaction duration of the first antibody and the second antibody were 2 hours and 1 hour, respectively. According to these optimal parameters, the standard linear equation, y = 0.0032x + 0.1769 (R^2^ = 0.9779, x, y representing MT concentration and OD_450_ value), was established for the determination of MT concentration with a valid range of 3.9–500 ng/ml. In verification experiments, the mean coefficients of variation of the intra-assay and inter-assay were 3.260% and 3.736%, respectively. According to the result of MT recovery, ELISA with an approaching 100% MT recovery was more reliable and sensitive than the Cd saturation assay. In conclusion, the newly developed ELISA of this study was precise, stable and repeatable, and could be used as a biomarker tool to monitor pollution by heavy metals.

## Introduction

Due to enhanced anthropogenic activities, cadmium (Cd) contamination of the aquatic environment has increased during recent years [1], [2], and is also frequently found in the Yellow River and Yangtze River of China [3], [4]. Studies showed that the background level of Cd in the Yangtze River water system was 0.019 μg/l [5]. Ma et al. [6] concluded that Cd level in the surface sediment of Dan River was just between the lowest effect level and severe effect level values, and could provide a serious ecological risk. Resulting toxicity from Cd on aquatic organisms is becoming a global environmental problem that results in the damage of aquatic ecosystems [7], [8]. In addition, Cd could cause an increase of reactive oxygen species that challenge the cellular antioxidant system, and lipid peroxidation in crustaceans [9].

In the defense against oxidative stress induced by Cd, MT represents an important intracellular antioxidant. MT metal-binding proteins with a low molecular weight and a high number of thiol groups have been reported in some species of fish and aquatic invertebrates [10–13]. High cysteine content enables MT to bind to particular heavy metals [14]. MT can associate with toxic metals (such as Cd and Hg), and is commonly involved in the detoxification of Cd and other non-essential metals [15]. Metal-induced MT, specifically Cd-MT, was found in many species, including the shore crab *Carcinus maenas* [16], and a freshwater and semi-terrestrial species *Potamon potamios* [17], suggesting the possibility that MT could be used as a biomarker in the monitoring of metal pollution in crabs generally. As reported by Matthiessen [18], MT as a core suit of biomarkers has been examined in the framework of biological effect quality assurance in monitoring programs that are also recognized at the European level. Despite serious river pollution in China, very few studies were focusing as yet on using MT as a biomarker.

Crustaceans are considered as suitable indicator species indicating environmental pollution in aquatic ecosystems because of their sediment-associated and less mobile life habits. Some crustacean species, such as the crayfish *Astacus leptodactylus* [19] and the crab *Carcinus maenas* [20] were chosen for the study of aquatic contamination. In the present study, the freshwater crab *Sinopotamon yangtsekiense* became the subject of our molecular approaches. This crab species is widely distributed in the Yangtze, the Huaihe River drainage basin, and the Yellow River Valley of China. In addition, laboratory studies by Ma and coworkers [21] showed that the MT level in tissues, especially hepatopancreas and gills, significantly increased after Cd exposure. The authors concluded that the crab can be used as a bioindicator organism for the monitoring of Cd contamination.

A traditional method of MT measurement is the Cd saturation method developed by Chen and Ganther [22]. Because of the high affinity between the SH group of MT to Cd, MT was obtained by quantitative determination of Cd content. However, this indirect determination could lead to an increase of error. Adverse effects can be intensified by extended exposure times to Cd. Another general method is provided by the enzyme-linked immunosorbent assay (ELISA). Because of its high sensitivity and specificity, ELISA has been used to detect traces of MT in some mammals, such as rats and rabbits [23]. Few studies, however, focused on the development of an ELISA to measure the MT content in crustaceans. Therefore, it is necessary to establish a more safe, convenient, sensitive, and efficient detection method for the monitoring of metal contamination in the environment. In the present study, we purified the MT of *S*. *yangtsekiense* through recombinant MT, exploiting the MT antiserum, and developed an ELISA to assess MT content in *S*. *yangtsekiense* and to monitor environmental Cd pollution.

## Materials and methods

### Ethics statement

The place where crabs were caught is privately owned. With the permission from the owner of the land, our study was carried out. All animal experiments were approved by the Shanxi Medical University Animal Research Platform Animal Ethics Committee. We also confirm that the current studies did not involve endangered or protected species. The work described in this article was performed in accordance with National and Institutional Guide-lines for the protection of animal welfare.

### Preparation of animals

Adult female freshwater crabs were purchased from Wulongkou fish market (Taiyuan, Shanxi Province, China), and were known to be caught from Qinghe River near Qingyan, Henan province in China. Before the experiments, female crabs were acclimated for three weeks in glass aquaria filled with dechlorinated tap water (pH 7.50 ± 0.13, temperature 20 ± 2°C). During acclimatization, the dissolved oxygen was kept at 8.0–8.3 mg/l, and the tanks were cleaned every two days.

### Construction of recombinant plasmids

Based on the cDNA sequence of *Sinopotamon yangtsekiense* MT gene, primers were designed using the Bioedit software (version 7.0.5.3). The sequences of two primers were as follows:

Upstream primer: 5′- GCCATGGCCCCTGATCCTTGCTGC- 3′

Downstream primer: 5′- AGGATCCGGG GCAGCAGGAGCAAG—3′

Restriction sites NcoI (upstream primer) and BamHI (downstream primer) underlined the primers. Using the cloning plasmid (pGEM-T-MT) constructed in a previous study (Ma et al., 2009) as a template, PCR was performed using 50 μl units including H_2_O 20 μl, 1020 s in 5 μl, Mg^2+^ 1.5 μl, BSA 1 μl, dNTP 5 μl, upstream primer 2.5 μl, downstream primer 2.5 μl, Pgem-T-MT10 μl, and *Taq* DNA polymerase 2.5 μl. Reaction was carried out with an initial denaturation step at 95°C for 5 min, followed by 30 cycles of 94°C for 30 s, 65°C for 30 s, 70°C for 1 min, and a final step of 70°C for 10 min. PCR fragments were detected by agarose gel electrophoresis, and extracted using DNA Gel Extraction Kit (Qiagen, Hilden, Germany). By T4 DNA ligase (New England Biolabs, Ipswich, MA), the extracted fragment digested with NcoI and BamHI was ligased to vector phoA at 4°C, and was transformed into DH5α competent cells. After sequencing the positive clones in order to ensure in-frame insertion, the recombinant phoA-MT vector was extracted and used to transform into *Escherichia coli* (*E*. *coli*) BL21 (DE3) strain (Novagen, Germany) for protein expression.

### Expression and purification of recombinant MT

The recombinant *E*. *coli* with phoA-MT strains was inoculated into Luria-Bertani (LB) media and grown at 37°C overnight with shaking at 250 rpm, and was transferred into fresh medium with low phosphorus (2.5 g/l peptone, 0.25 g/l yeast extract, 3g/l (NH_4_)_2_SO_4_, 5g/l NaCl, 1g/l MgSO_4_, 4 g/l glucose, 30 μM/l ZnSO_4_) to induce exponential growth until the phosphorus exhaustion. After collection at 5,900 g for 25 min (Eppendorf 5415R, Hamburg, Germany), 1 g fresh *E*. *coli* was added with 5 ml lysate (20 mM Tris, 500 mM NaCl, 1% Trionton, 10 mM imidazole pH 7.8), and was stirred with an intermitted ultrasonic (360 ~ 400 w) treatment for 30 min in the ice bath. The homogenate was centrifuged by a refrigerated centrifuge for 15 min at 12,000 g at 4°C. The clear supernatant was removed and filtered through a 0.22 μm filter. By Ni-NTA affinity chromatography, the final sample was run over a Ni-NTA Superflow resin (Qiagen Inc., Hilden, Germany) to separate the His-tagged MT from other proteins following 50 mM and 150 mM imidazole. The separated His-tagged MT was concentrated by ultrafiltration in centricon-30 filters (3,000 MWCO filter; Amicon), and then was stored in a storage medium (100 mM Tris-HCl; 0.2 mM phenylmethanesulfonyl fluoride; 10mM DL-dithiothreitol; pH 8.6).

### Recombinant protein identification by western blotting

Separation was conducted on 16.5% polyacrylamide gels in the presence of 0.1% Sodium dodecyl sulfate (SDS) dissolved in 1.5 M Tris-glycine, (pH 8.8). Proteins separated by sodium dodecyl sulfate-polyacrylamide gel electrophoresis (SDS-PAGE) were transferred onto polyvinylidene difluoride (PVDF) membranes incubated with PBST buffer (pH 8.0) containing 5% nonfat dry milk to stand overnight at room temperature. After the membranes were finally washed 4 × with PBST for 10 min, the expressed proteins were probed with either monoclonal anti-His-tag obtained from rabbits immunized against individual 6 his-MT-fusion proteins (Rockland Immunochemicals, Gilbertsville, PA) by incubating overnight at 4°C. After the membranes were washed 4 × with PBST buffer for 5 min, they were incubated with a secondary antibody (Horseradish Peroxidase-conjugated goat anti-rabbit IgG, Rockland Immunochemicals, Gilbertsville, PA) for 2 hours. The membranes were finally washed with 4 × with PBST buffer for 10 min before the chemiluminescent substrates of a western blot were applied with DAB Horseradish Peroxidase Color Development Kit (Roche, Mannheim, Germany).

### Preparation of antiserum and immunoblotting

Through dialysis using a 3,000 MWCO membrane (Millipore Corp., Bedford, MA, USA) against 0.01 M Tris-HCl (pH 7.8) containing 0.2 mM PMSF, imidazole was eliminated from the purified protein. Then the recombinant MT mixed with an equal volume of QuickAntibody (Kangbiquan Inc., China) was injected into the hind leg muscle of (1 ml per mouse) of the animals. After three weeks, another injection using QuickAntibody adjuvant was given. At the 5th week, blood was collected from the eyeball, and antiserum was centrifuged at 3,000g for 5 min. After removing precipitated residues, the blood serum to which 0.02% NaN_3_ was added was stored at -80°C. The MT concentration in the tissue was determined using the method of Bradford [24] where bovine serum albumin was used as a standard.

By SDS-PAGE, proteins were separated and transferred onto PVDF membranes incubated with PBST buffer (pH 8.0) containing 5% nonfat dry milk to stand overnight at room temperature. After the membranes were finally washed 4 × with PBST for 10 min, the expressed proteins were probed with monoclonal anti-MT obtained from mouse by incubating overnight at 4°C. After the membranes were washed 4 × with PBST buffer for 5 min, they were incubated with a secondary antibody (goat anti-mouse IgG conjugated with alkaline phosphates, Sangon, China) for 2 hours. The membranes were finally washed with 4 × with PBST buffer for 10 min before the chemiluminescent substrates of western blot were applied with DAB Horseradish Peroxidase Color Development Kit (Roche, Mannheim, Germany).

### Development of enzyme-linked immunosorbent assay (ELISA)

#### Comparison between direct coating ELISA and antibody sandwich coating ELISA

The analysis of direct coating ELISA was carried out according to the method of Chen et al. [25]. Polystyrene microtiter plate (Corning, USA) was coated with purified MT. Liquid was discarded after overnight incubation at 4°C. By incubating the plate with PBST containing 1% bovine serum albumin (BSA) for 2 h at 37°C, the nonspecific binding sites were blocked. Anti-MT polyclonal antiserum diluted to 1:20,000 with 1% BSA was added and incubated for 2 h at 37°C. After being washed with PBST, the plate was incubated for 1 h with goat anti-mouse IgG-HRP (Sigma, USA) diluted to 1:2,000 with the 1% BSA. TMB chromogenic reagent (Solarbio, Beijing, China) was added to the plate after washing with buffer. After 50 ml of 2 M H_2_SO_4_ was added to plots to stop the reaction, the absorbance was measured at 450 nm by spectrophotometer (SpectraMax M5, Molecular Devices Corp., San Francisco, CA, U.S.A.).

Except coating the plate twice with antibody, the main steps of antibody sandwich coating ELISA was similar to the procedure of directly coated ELISA. First, the plate was coated with anti-MT polyclonal antiserum. After incubation in 1% BSA, purified MT was added to the plate for the first reaction. Then the plate was coated in goat anti-mouse IgG-HRP for the second reaction.

#### Optimization of reaction conditions

With the directly coated ELISA, the potency of MT antibody was detected in the samples. Firstly, a polystyrene microtiter plate was coated with 1μg/ml purified MT, and anti-MT polyclonal antiserum was diluted to different concentrations (1: 1,000 1: 5,000 1:10,000 1:20,000 1:40,000 1:80,000 1:160,000) with PBST. The other steps were the same to those of the directly coated ELISA.

Using checkerboard titration, the optimum concentration of antibody and MT (antigen) was determined. Different concentrations of MT (0, 7.8, 15.7, 31.25, 62.5, 125, 250, 500 and 1000 ng/ml) were transversely added to 96-well polystyrene microtiter plates, and different dilution MT antisera (1:40,000 1:60 000 1:80 000 1:10,000 1:120,000 1:140,000 1:160,000) were longitudinally added. The other steps were similar to samples directly coated by ELISA. According to the result of the checkerboard method ELISA, the standard curve with different MT antibody (1:120,000, 1:140,000, 1:160,000) was further compared to determine the optimal dilution of MT antibodies. In addition, the effect of different coating times, and the reaction times of MT antibodies with goat anti-mouse IgG-HRP on absorbance values was detected to optimize various ELISA parameters.

#### Preparation of standard curve

Based on the above experimental results, the standard curve was prepared. MT concentrations included 1.95, 3.90, 7.80, 15.63, 31.25, 62.50, 125.00, 250.00, 500.00, 1,000.00 and 2,000.00 ng/ml. Optimized ELISA parameters were used for the measurement. According to the linear relationship and the coefficient of variation, the MT concentration range showed good repeatability, and mass concentration correlation was chosen. Using the multiple mass concentration gradient of ELISA a standard curve was drawn.

### Quantification of MT in crab hepatopancreas with optimized ELISA method

Four freshwater crabs were randomly selected. Before sampling, crabs were anesthetized by placing them on ice for 15 min. After dissection of the cephalothorax, hepatopancreas were immediately sampled. Hepatopancreas tissue (0.1 g) was homogenated in 0.9 ml precooled homogenization buffer, and the homogenate was centrifuged (4°C, 12,000g) for 15 min. The supernatant was used for MT measurement by ELISA, and each sample was repeated in four wells. The experiment was repeated four times with the same experimental procedure. Finally, the standard deviation and coefficient of variation about inter-assay and intro-assay was analyzed to verify the reliability and stability of the established ELISA method.

### The recovery rate of MT by ELISA and Cd saturation method

Accuracy, linearity, and sensitivity of the ELISA and Cd saturation assay were investigated using *in vitro* prepared MT. The recovery of different MT (62.5, 125, 250, 500, 1,000 ng) was determined with ELISA according to the above optimal parameters and Cd saturation assay following the methods of Ma et al. [21]. To investigate possible influences of biological matrices on the reliability of both methods, recovery of different MT (62.5, 125, 250, 500, 1000 ng) were added to the crab hepatopancreas homogenate (180 ng MT), and the detection level of both assays were compared.

### Statistics

All data were represented by means ± standard deviation (S.D.), and statistical computations were performed with SPSS 17.0. Levine’s test of the one-way analysis of variance was used for the homogeneity of variance, and Tukey’s method for multiple comparisons. By *T-*Test (independent samples *T-*Test), differenced of the different MT concentration, MT antisera, and coated methods were evaluated, and probability values less than 0.05 were considered as significant.

## Results

### Expression, purification of MT and identification by western blotting

**Fig 1:**
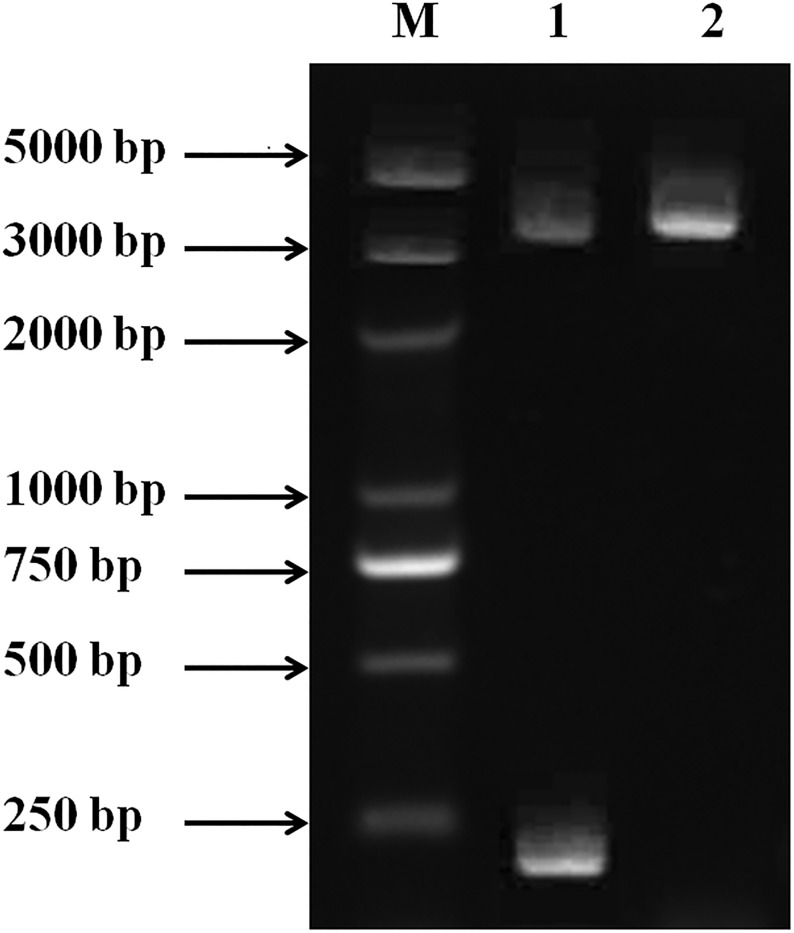
Identification of the recombinant plasmid phoA-MT by restriction enzyme digestion and PCR. 1. DNA marker; 2. phoA-MT digested with NcoI and BamHI endonucleases (Up: Phoa plasmid, Down: MT); 3. Recombinant plasmid phoA-MT.

Fig 1 shows the results of recombinant plasmid phoA-MT before and after digestion by NcoI-BamHI. A single nucleic acid band was detected in lane 2 (phoA-MT) with a molecular mass of 3300 bp, and another two nucleic acid bands were also detected in lane 1 (phoA-MT digested with NcoI-BamHI), respectively with a molecular mass of 3000 bp and 200bp, which were substantially consistent with the theoretical molecular mass of the phoA and MT, respectively. Moreover, DNA sequencing in Sangon Biotech Co. Ltd (China) confirmed the phoA-MT expression plasmid, and that the MT gene was cloned in the correct frame after the phoA coding sequence.

**Fig 2:**
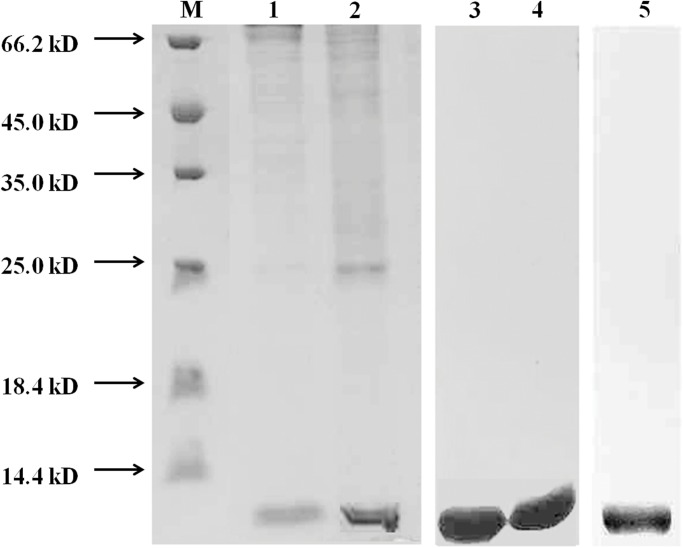
SDS-PAGE and western blot analysis of purified recombinant MT. M: protein molecular weight markers; lane 1: MT separated with 150 mM imidazole; lane 2: MT separated with 50 mM imidazole; lane 3 and 4: Western blot of recombined MT with anti-6His antibody; lane 5: Western blot of recombined MT fusion protein with purified MT antiserum.

MT extract was fractionated by SDS-PAGE, and protein bands were detected with a molecular mass of 7 kDa (lane 1 and 2, Fig 2). The purification and separation of expressed protein was carried out with different concentrations of imidazole by Ni-NTA affinity chromatography, and a better separation with 150 mM imidazole (lane 1, Fig 2) could be observed than that of 50 mM imidazole (lane 2, Fig 2). The expressed and purified proteins were identified by western blotting. The monoclonal anti-His-tag obtained from rabbits immunized against individual 6 his-MT-fusion proteins and horseradish peroxidase-conjugated goat anti-rabbit IgG were used as the first and secondary antibody, respectively. As shown in lane 3 and 4 of Fig 2, two color bands at about 7 kDa were observed, which was consistent with our expectations, and protein mass spectrometry in Sangon biotech Co. Ltd (China) also proved that the purification protein was MT of *S*. *yangtsekiense*.

### Specificity of anti-MT serum

The specificity of the anti-MT serum was determined by Western blotting. As shown in lane 5 of Fig 2, when the purified MT was used, only one major band was detected, suggesting that the antiserum was specific for MT. Cross-reactivity was negligible.

### ELISA of MT

#### Results of direct coating ELISA and antibody sandwich coating ELISA

In Table 1, the values of OD_450_ between MT concentration groups measured from samples directly coated by ELISA showed significant differences, while significant differences was not observed between the three MT concentration groups (1000, 500 and 250ng/ml) in antibody sandwich coating ELISA. For the same MT level group, OD_450_ of the former method was higher than that of the latter one. The average coefficient of variation, however, was opposite, suggesting a better repeatability in samples by direct coating ELISA. The above results suggested that direct coating ELISA was more suitable than antibody sandwich coating ELISA for MT determination in *S*. *yangtsekiense*.

**Table 1:** Comparison of OD_450_ between the samples direct coating ELISA and the anti-body sandwich coating ELISA.

MT concentration (ng/ml)	Sample direct coating (n = 3)		Anti-body sandwich coating (n = 3)	
	OD_450_ (mean ± S.D.)	Coefficient of variation (%)	OD_450_ (mean ± S.D.)	Coefficient of variation (%)
**2000**	3.58 ± 0.05^a^	1.28	0.52 ± 0.04 ^a^*	8.6
**1000**	2.94 ± 0.13^b^	4.44	0.48 ± 0.01 ^b^*	3.03
**500**	2.26 ± 0.07^c^	3.26	0.36 ± 0.03 ^b^*	8.85
**250**	1.80 ± 0.06^d^	3.19	0.34 ± 0.01 ^b^*	4.4
**125**	1.36 ± 0.01^e^	2.99	0.32 ± 0.04 ^c^*	12.21
**62.5**	1.10 ± 0.08^f^	2.97	0.27 ± 0.01 ^c^*	4.71
**Mean**		3.53		6.97

Values within a column having different superscript letters are significantly different and values within a line with “*” are significantly different (*p* < 0.05). N means sample number.

#### Optimization of reaction conditions

Based on the above conclusion, the most suitable dilution factor of MT anti-serum was detected by direct coating ELISA. Table 2 showed the effect of different dilution factors on the OD_450_. We observed that the coefficient of variation was less than 5% when the dilution factor of MT was 40,000–160,000. Owing to the higher potency of MT antibody, this indicated that a 1:160,000 dilution factor of MT anti-serum could be used for detection of MT.

**Table 2:** The OD_450_ of ELISA by the different dilution rates of MT antiserum.

MT antisurem dilution rate	OD450 (mean ± S.D, n = 3)	Coefficient of variation (%)
**1:1,000**	3.41 ± 0.27	7.91
**1:5,000**	3.29 ± 0.19	5.78
**1:10,000**	3.22 ± 0.28	8.7
**1:20,000**	3.19 ± 0.22	6.9
**1:40,000**	2.96 ± 0.10	3.38
**1:80,000**	2.33 ± 0.10	4.29
**1:160,000**	1.75 ± 0.08	4.57
**BSA**	0.09 ± 0.01	1.02

Values within a column having different superscript letters are significantly different and values within a line with “*” are significantly different (*p* < 0.05). N means sample number.

**Fig 3:**
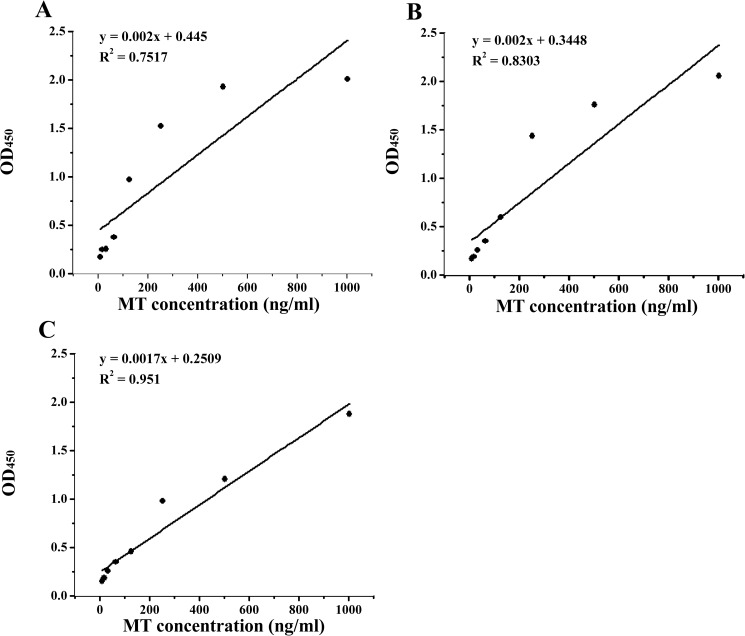
Regression curve of ELISA between MT concentration and OD_450_ with 1:120000 (A), 1:140000 (B), and 1:160000 (C) dilution of MT anti-serum.

By checker-board titration method, the most suitable concentration of MT and MT anti-serum was shown in Table 3. Determination standards of the most suitable working concentration was that OD_450_ of positive well was about 1, and the negative control wells were less than 0.1. As shown in Table 3, MT antibody dilution factor in 1:120,000, 1:140,000 and 1:160,000 values could meet the standard of judgment, and the correlation between the OD_450_ and coated MT concentration was better. Therefore, it was necessary to analyze the regression curve of diluted MT-antibody 1:120,000, 1:140,000, and 1:160,000. For the same MT concentration, the value of MT-antibody dilution groups (1:120,000, 1:140,000) was higher than that of 1:160,000, while correlation of the regression curve at 1:160,000 was higher than that of the former two groups (Fig 3A–3C). Therefore, MT antiserum dilution factor was 1:160,000 under the present experimental conditions.

**Table 3:** The OD_450_ of the checkerboard titration of different concentration combinations of MT anti-body and MT.

MT antiserum dilution rate	MT coating concentration (ng/ml)								
	0	1000	500	250	125	62.5	31.25	15.7	7.8
**1:40,000**	0.071	3.009	2.586	2.124	1.122	0.985	0.411	0.493	0.34
**1:60,000**	0.079	3.098	2.01	1.808	1.216	0.606	0.44	0.406	0.206
**1:80,000**	0.083	2.338	2.001	1.675	1.024	0.425	0.277	0.3	0.144
**1:100,000**	0.052	2.245	1.943	1.585	0.892	0.371	0.269	0.205	0.181
**1:120,000**	0.044	2.011	1.931	1.525	0.974	0.376	0.254	0.248	0.17
**1:140,000**	0.041	2.057	1.759	1.437	0.6	0.348	0.259	0.191	0.168
**1:160,000**	0.032	1.879	1.205	0.981	0.458	0.352	0.259	0.186	0.151

Table 4 showed the results of different coating periods on OD_450._ Significant differences could be observed during 8 hours compared with to 12 and 16 hours. There was no significant difference between 12 and 16 hours. The coating period of 12 hours seemed to be suitable for our experiment.

**Table 4:** The effects of different coating periods on OD_450_.

Coating period (hour)	MT coating concentration 500 ng/ml		MT coating concentration 250 ng/ml	
	OD_450_ (mean ± S.D., n = 3)	Coefficient of Variation (%)	OD_450_ (mean ± S.D., n = 3)	Coefficient of Variation (%)
**8**	0.44 ± 0.08^a^	18.18	0.34 ± 0.08^a^	12.36
**12**	1.14 ± 0.11^b*^	3.82	0.84 ± 0.12^b^	2.83
**16**	1.28 ± 0.12^b*^	4.16	0.96 ± 0.11^b^	3.22

Values within a column having different superscript letters are significantly different and values within a line with “*” are significantly different (*p* < 0.05). N means sample number.

In Table 5, OD_450_ of two concentrations MT, OD_450_ showed an upward trend with the extension of the reaction time. However, no significant difference between 3 hours and 4 hours was observed. After 3 hours of the first antibody reaction, the variation coefficient of 500ng/ml and 250ng/ml MT group was greater than 5%, which did not meet the requirements. At the same time, there was no significant difference between the two MT groups after 0.5 h reaction, and the coefficient of variation in both MT groups was greater than 5%. According to the above results and analysis, we consider that the best reaction time of the first antibody was 2 hours.

**Table 5:** The effects of different reaction periods of the first antibody on OD_450_.

Reaction period of the first antibody (hour)	MT coating concentration 500 ng/ml		MT coating concentration 250 ng/ml	
	OD_450_ (mean ± S.D., n = 3)	Coefficient of Variation (%)	OD_450_ (mean ± S.D., n = 3)	Coefficient of Variation (%)
**0.5**	0.19 ± 0.02^a^	7.94	0.15 ± 0.03^a^	21.56
**1**	0.38 ± 0.08^b*^	20.71	0.18 ± 0.02^b^	12.35
**2**	1.11 ± 0.04^c*^	3.79	0.95 ± 0.03^c^	3.26
**3**	1.63 ± 0.13^d^	8.15	1.45 ± 0.39^d^	26.69
**4**	1.69 ± 0.10^d*^	5.89	1.34 ± 0.15^d^	11.07

Values within a column having different superscript letters are significantly different and values within a line with “*” are significantly different (*p* < 0.05). N means sample number.

### Standard curve

**Fig 4:**
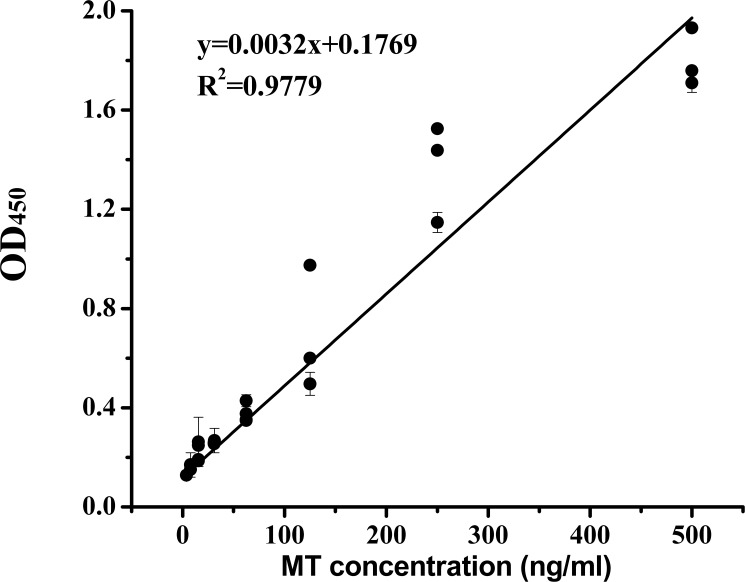
The ELISA standard curve for purified MT. Data are expressed as mean ± standard deviation (n = 5).

According to the optimized ELISA of sample direct coating ELISA, OD_450_ coating with different MT concentrations was measured. As shown in Table 6, the OD_450_ of 3.90 ng/ml MT was higher than that of controls, and the difference was significant (*P* < 0.05). Therefore, the lowest MT concentration detected by the current ELISA was 3.90 ng/ml. With an increase of MT concentration, the values of OD_450_ were also increasing. However, the significant different between the 500.00, 1 000.00, and 2 000.00 ng/ml MT concentration group was not observed. Based on the above results, a standard curve within the range of 3.90 and 500.00 ng/ml was drawn. From Fig 4, we observed an optimal and significant linear relationship between the MT concentrations and OD_450_ (y = 0.0032x + 0.1769, R^2^ = 0.9779, y: OD_450,_ x: MT concentration). Therefore, the working range of ELISA was from 3.90 to 500 ng/ml.

**Table 6:** The OD_450_ and its coefficient of variation of a serial of MT concentration.

MT concentration (ng/ml)	OD450 (mean ± S.D., n = 3)	Coefficient of Variation (%)
**Control**	0.095 ± 0.004	3.68
**1.95**	0.119 ± 0.005	4.22
**3.9**	0.128 ± 0.003	2.38
**7.8**	0.169 ± 0.049	9.08
**15.7**	0.262 ± 0.100	8.01
**31.3**	0.268 ± 0.049	8.23
**62.5**	0.428 ± 0.024	5.62
**125**	0.896 ± 0.046	5.13
**250**	1.447 ± 0.040	2.79
**500**	1.710 ± 0.039	2.25
**1000**	1.725 ± 0.033	1.89
**2000**	1.558 ± 0.099	6.35

### Quantification of MT in crab hepatopancreas with optimized ELISA method

According to optimized reaction conditions of ELISA and the standard curve, four freshwater crabs were randomly selected and the hepatopancreas was used for validation experiments. As shown in Table 7, the average coefficient of variation within the samples and between samples was 3.26% and 3.726%. The results were in accordance with the requirements of less than 5%. Moreover, no significant difference was observed within the samples and between samples. Therefore, the ELISA determination method established in the present experiments was stable and repeatable.

**Table 7:** The MT contents in the hepatopancreas of *Sinopotamon yangtsekiense*.

Sample	Intra-assay		Inter-assay	
	MT concentration (μg/mg protein)	Cofficient of variation (%)	MT concentration (μg/mg protein)	Coefficient of variation (%)
**1**	0.209 ± 0.005	2.4	0.212 ± 0.007	3.324
**2**	0.212 ± 0.003	1.5	0.210 ± 0.004	1.873
**3**	0.213 ± 0.013	6.222	0.214 ± 0.009	4.287
**4**	0.218 ± 0.006	2.918	0.215 ± 0.012	5.462
**Mean**		3.26		3.736

### The recovery rate of MT

**Fig 5:**
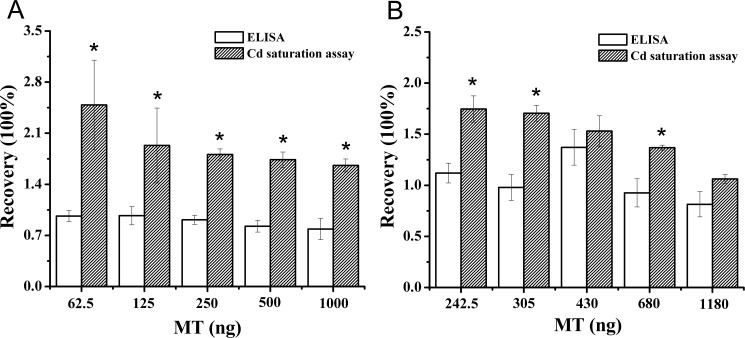
**Recovery of purified MT (A) and MT added to crab hepatopancreas cytosol (B) as determined with the ELISA and Cd saturation assay.** Data are expressed as mean ± standard deviation (n = 5). Comparison between the ELISA and Cd saturation assay groups is notified as * *p* < 0.05.

As showed in Fig 5A, ELISA showed an excellent fit with the expected values of 62.5–500 ng MT, whereas MT reproducibility decreased with a tendency to underestimate the MT content at 1000 ng. However, the Cd saturation assay showed a tendency to overestimate the MT content, especially the 62.5 ng MT.

Because of the possible influences of biological matrices on the reliability of both methods, it was necessary to determine the recovery of MT added to the hepatopancreas homogenate. As illustrated in Fig 5B, the Cd saturation assay significantly overestimated the theoretical values with a decrease of MT contents. In contrast, better results were obtained with the ELISA compared to the Cd saturation assay, showing better agreement with the theoretical values.

## Discussion and conclusion

The present study extends our previous research for the development of biomarkers for metal exposure in the freshwater crab *Sinopotamon yangtsekiense*. With a series of analysis using molecular biochemical approaches, we demonstrated that MT from this crab was ideal for mRNA sensitivity, protein activity, and antioxidant defense potential upon diverse environmental conditions and pollutant exposures [9], [21], [26]. To check its usefulness as an indicator for marine contaminants, we developed polyclonal antibodies and ELISA platform MT protein from this crab.

As for other invertebrate ELISA approaches using polyclonal antibodies, Volz and Chandler [27] reported a low detection limit (1.9 ng/ml) and a broad working range from 31–1,000 ng/ml using the VTN polyclonal antibody-based ELISA in the marine meiobenthic harpacticoid, *L*. *plumulosus*. In the Chinese mitten-handed crab, *Eriocheir sinensis*, Chen et al. [25] reported a similar working range of 8–500 ng/ml using the VTN polyclonal antibody-based ELISA. Ghekiere et al. [28] showed that the working range of mysid VTN ELISA was 4–500 ng/ml using VTN polyclonal antibody. Arcos et al. [29] developed a VTN polyclonal antibody-based ELISA system with a working range 10–400 ng/mg in the oyster, *Crassostrea corteziensis*. Therefore, we suggest that the crab MT ELISA was sufficiently sensitive as compared to working ranges of previous publications.

Crustacean MT from a number of species has been recently characterized. MT in many species showed a native molecular mass ranging from 3–15 kDa, such as in the shore crab [16], and the blue crab [30]. In this study, MT has been produced and purified from *E*. *coli* by the phoA-fusion technology, gel filtration chromatography and anion-exchange chromatography. By sodium dodecyl sulfate-polyacrylamide gel electrophoresis (SDS-PAGE) and time-of-flight mass spectrometry analysis, we found that *S*. *yangtsekiense* MT existed as monomer forms. A monomer molecular weight of 7 KDa was determined which was within the range found in other species. These results suggested that we have purified a single MT isoform. It is in agreement with the observations in the freshwater and semiterrestrial species *Potamon potamios* [17]. However, other studies in crustaceans found different forms, such as in the mud crab *S*. *serrata* [31].

A polyclonal MT antiserum was raised against a purified MT of *S*. *yangtsekiense* which specifically recognized MT from *E*. *coli*. Cross-reactions were not a problem with the other proteins. The observed high specificity indicated that the polyclonal MT antiserum may be useful for various immunological studies of MT, and also allowed us to develop an ELISA to quantify the MT concentrations. The ELISA was sensitive for *S*. *yangtsekiense* MT with a working range of 3.9–500 ng/ml. Chan et al [32] developed an ELISA for the blue crab (*Callinectes sapidus*) based on polyclonal antibodies that had a sensitivity of 10 ng, which is comparable to the present ELISA. Moreover, the ELISA for *S*. *yangtsekiense* has a lower and acceptable inter-assay and intra-assay coefficient of variation. The precision of the assay is sufficient to allow a direct comparison of samples on the same plate and from different plates assayed at different times. In order to validate the applicability of the ELISA for different concentrations of MT, we established a standard curve using diluted samples. The dilution curve for hepatopancreas homogenate was similar to the non-diluted ELISA standard curve. Thus, the ELISA is suitable for quantification of MT in hepatopancreas where MT is expected to be at similar concentrations.

Currently, several methods for the quantification of MT, such as Ag saturation, Cd saturation, and enzyme-linked immunosorbent assay, were developed and are readily available [33–37]. Bienengräber et al [37] found a difference between methods, and suggested that enzyme-linked immunosorbent assay were more reliable and sensitive, which was consistent with our studies. According to the result of MT recovery, a higher standard error at lower MT concentrations and an overestimated MT recovery was observed in the Cd saturation assay. The situation was more serious in the recovery of MT added to the hepatopancreas homogenate. During Cd saturation assay, heat treatment causing a loss of heat-labile MT and excessive hemoglobin stripping off Cd from Cd-thionein may lead to a unreliable consequence [38], [39]. An overestimation of the recovery of MT added to the hepatopancreas homogenate may be explained by other low-molecular-weight Cd-binding components in the cytosol. Based on the reliable quantification of MT, immunological methods required appropriate characterization of the antibodies, the ELISA accurately determined MT in the presence and absence of hepatopancreatic cytosol. With a detection limit of about 40 ng MT and an approaching 100% MT recovery, this assay was more sensitive and reliable than the Cd saturation assay. Our immunological approach was sufficiently sensitive to quantify MT, suggesting that MT could be used as a biomarker to monitor pollution by heavy metals.

To produce MT in large quantities for basic research and the development of an MT anti-serum, it is important to produce pure protein that retains biological activity. In the present study, recombinant metallothionein (MT) from the freshwater crab (*Sinopotamon yangtsekiense*) has been successfully produced by *E*. *coli* using the phoA-fusion technology. In addition, the biologically active MT from phoA-fusion enhancing expression and purification was confirmed by western-blotting. Based on the purified MT and MT anti-serum, the reaction parameters of an enzyme-linked immunosorbent assay (ELISA) was developed. According to the results of verification and recovery experiments, the presently developed ELISA showed more reliable, sensitive and repeatable results. We suggest that the MT crab ELISA provides a suitable tool to quantify the biochemical effects of contaminants by detection of MT synthesis. A high purity metallothionein and anti-metallothionein ELISA could further find important applications in biomonitoring programs, for example, in the development of a new generation of sensors indicating metal exposure.
